# Overcoming the Crisis of the Reviewing Process: Responsibility of a Scientific Journal

**DOI:** 10.3390/tomography8010043

**Published:** 2022-02-18

**Authors:** Emilio Quaia, Filippo Crimì, Elisa Baratella

**Affiliations:** 1 Department of Radiology, University of Padova, Via Giustiniani 2, 35128 Padova, Italy; filippo.crimi@unipd.it; 2 Department of Radiology, University of Trieste, 34134 Trieste, Italy; elisa.baratella@gmail.com

Manuscript reviewers and the accuracy of the review process are fundamental to the quality of a scientific journal. Authors entrust their research to reviewers and should expect the highest ethical standards and expertise in the reviewing process of their scientific work. Moreover, authors place tremendous confidence in peer reviewers’ confidentiality and professionalism, since manuscript submissions are unpublished material; as such, a degree of trust is established when an author submits their paper to a scientific journal. Reviewers’ comments should be constructive, honest, and polite, and should not be influenced by commercial interests, personal relationships, or agendas. Even findings that are not statistically significant or report negative associations should not influence reviewers. Scientific journals can rely on hundreds of registered reviewers who are generally classified based on keywords reflecting their field of expertise. At *Tomography*, we are fortunate to have more than 300 active reviewers with almost 200 manuscripts submitted in 2021 for a total of 70 papers published in the same year.

A reviewer assigns a numerical score to a manuscript to quantify its estimated merit for publication. Importantly, a reviewer does not select a manuscript for publication but, instead, simply renders an opinion. This opinion is weighed against the opinion of one or more additional reviewers and the editor in chief finally makes a decision about a manuscript’s potential publication. The final decision remains the prerogative of the academic editor, who scores the reviewer’s assessment of the manuscript.

A careful evaluation of the submitted manuscript implies the assessment of its scientific merit and its ranking compared to the existing literature [1]. Reviewers are charged with the essential task of judging whether a manuscript is important, scientifically valid, coherent and readable, and appropriate for a particular journal. Who else could stop the progression of a flawed manuscript or facilitate the rise of an otherwise unseen study? Only an experienced and accurate reviewer can do that. Of course, not all reviewers are equally skilled in this task. But who are the best reviewers? A number of characteristics, including age, subspecialty, number of years reviewing, academic rank, and type of practice (academic or private) could be related to the quality of a reviewer [2]. Editors typically select experts in the subject to review the manuscript. More experienced reviewers or those with a higher academic rank may underestimate the importance of manuscripts [3], while newer reviewers may express a more objective assessment of a manuscript. Generally, there are no fixed criteria that define an accurate and dedicated reviewer, since many confounding factors can impact their task, including the reviewer’s level of expertise in the field of the manuscript’s topic, the time the reviewer can dedicate to the manuscript according to the specific period of manuscript handling, the potential competition between reviewers and authors in a particular scientific field, etc.

Journals differ in their peer review requirements and guidelines, such as in the number and type of reviewers selected for each manuscript. Editors may further improve the average quality of their published manuscripts by selecting reviewers who are well grounded in the scientific method and who have knowledge of some statistical techniques. However, the key issue is that the role of the reviewer is now facing a real crisis. Reviewing is a time-consuming process and the increasing pressure to publish may make reviewers less available to dedicate time to assessing the work of their colleagues. The increasing number of journals has caused a steep increase in reviewing invitations to individuals who frequently serve as reviewers for many scientific journals. Moreover, the reviewer’s work goes largely unseen, even though the success of peer review hinges on the skill, discernment, dedication, and fair-mindedness of a large coterie of expert reviewers. Unfortunately, no impact factor or H index increase is related to good peer review.

There are two essential components in the reviewing process: knowledge of the discipline and dedication to the review process, both of which can be provided by the reviewer. Although the reviewer could be highly knowledgeable in a particular field, their dedication to the review process of a particular manuscript could be even more important, and it is not a complete certainty that the reviewer will dedicate the necessary time to a manuscript to ensure an accurate and objective assessment. Establishing a system to select appropriate reviewers and to improve the quality of reviews is the basic and essential responsibility of the journal.

Since the quality of reviews is of the utmost importance to a journal’s scientific value, we ask whether there is a strategy that could improve this. In our opinion, there are, essentially, eight items that could improve the quality of reviews.

1. The highest accuracy in selecting keywords from reviewers. Each reviewer should spend some time selecting those keywords that best represent their knowledge, and they should continuously update these keywords according to their professional development. Keywords should not only include imaging techniques, but also specific disease features.
2. When a reviewer becomes too busy or no longer appreciates being involved in the review process, they should stop their participation. Moreover, when a reviewer refuses many invitations, the journal editor should mark their status as inactive.
3. No one can know everything, even in their own field. For this reason, the reviewer responsible for a particular manuscript should be allowed to involve other individuals—especially those considered potential future readers, although not those on the reviewer board—in the review process. This might be useful when the topic of a manuscript is very specific, even if its content aligns with the reviewer’s academic expertise.
4. Editors should not proceed with reviewer involvement when the manuscript is manifestly unsuitable for publication. This will save the reviewer time and allow them to focus on manuscripts worthy of undergoing the reviewing cycle.
5. The editor should involve at least three reviewers for each manuscript. This will improve the objective assessment of the scientific value of each manuscript.
6. The best reviewers should have the highest visibility. Moreover, we do not consider publication of reviewer names to be sufficient. A public database with dedicated scores (e.g., number of reviewed papers and scores) should provide the best reviewer names, similarly to databases that provide authors’ bibliometric scores (e.g., Scopus and Web of Science). Of course, this implies the accurate scoring of reviewers by journal editors. A further solution could be a suitable salary provided by journals for those reviewers who perform best in the reviewing process.
7. Editors should pay more attention to reviewers’ criticisms than to reviewers’ scores. Reviewer scores do not reflect the true scientific value of a manuscript. Only reviewer comments help the editor to make the right decision, and they also aid authors in improving the scientific value of their manuscript.
8. Journals should not overwhelm reviewers with more than three manuscripts each year. An excessive number of papers to be refereed does not grant enough time to dedicate to each paper.
